# Malignant pleural effusion facilitates the establishment and maintenance of tumor organoid biobank with multiple patient-derived lung tumor cell sources

**DOI:** 10.1186/s40164-024-00581-9

**Published:** 2024-11-15

**Authors:** Lingwei Wang, Yanli Yu, Yanhua Fang, Yanjiao Li, Weiting Yu, Zhe Wang, Jinyan Lv, Ruoyu Wang, Shanshan Liang

**Affiliations:** 1 The Key Laboratory of biomarker high throughput screening and target translation of breast and gastrointestinal tumor, Affiliated Zhongshan Hospital of Dalian University, No.6 Jiefang Street, Zhongshan District, Dalian, 116001 Liaoning China; 2 Department of Thoracic Surgery, Affiliated Zhongshan Hospital of Dalian University, No.6 Jiefang Street, Zhongshan District, Dalian, 116011 China; 3 Oncology Department, Affiliated Zhongshan Hospital of Dalian University, No.6 Jiefang Street, Zhongshan District, Dalian, 116001 Liaoning China

**Keywords:** Patient-derived organoids, Lung cancer organoids, Malignant pleural effusion, Airway organoids, Proliferation, Drug sensitivity

## Abstract

The Patient-Derived Organoids (PDOs) has demonstrated significant potential in personalized medicine. However, the initial establishment of lung cancer organoids (LCOs), and timely therapeutic recommendations face several challenges. Particularly, the current culture systems have not yet achieved the capability to long-term cultivation of all lung tumor sample sources, including malignant pleural effusion (MPE), which poses significant barriers to the rapid clinical translation of PDOs. Here, we established a LCOs biobank derived from various tumor cell origins and investigated the impact of supplementing culture media with MPE supernatant on organoid formation, culture duration, and drug sensitivity. Our findings indicate that MPE can enhance the successful rate of LCOs by extending the passage number and promoting the initial formation of difficult-to-culture samples, such as those derived from MPE or cell lines that were previously unsuccessful in Airway Organoid (AO) medium. MPE also facilitates the rapid proliferation of LCOs, reducing the culture duration by over 50%. Additionally, LCOs exhibit increased chemoresistance in the presence of MPE, which modifies stem cell distribution and reshapes the internal structure of the organoids. Overall, this study highlights the significance of MPE in facilitating the establishment and maintenance of LCOs, and its potential for translational applications in lung cancer research and personalized.

**Supplementary Information:**

The online version contains supplementary material available at 10.1186/s40164-024-00581-9.

**To the editor**,

Lung cancer organoids (LCOs), derived from tumor tissues or malignant serous effusion, exhibit strong correlations between drug sensitivity and clinical responses, underscoring their potential in personalized medicine and their value as a research model [1–4]. The current AO (airway organoid) media culture system [5] is inadequate for the long-term culture of LCOs derived from challenging samples such as malignant pleural effusion (MPE). The prolonged time required to establish PDOs also limits the use of PDO-based drug testing in clinical settings [2, 6]. The MPE supernatant is rich in serum proteins [7], as well as growth factors and cytokines associated with pro-inflammatory, oncogenic, and angiogenic properties [8], suggesting it provides a microenvironment that supports tumor growth. However, the influence of MPE on the formation and differentiation of LCOs remains largely unexplored.

We established a total of 39 LCOs and 5 normal lung tissue organoid lines from surgically resected tumor tissues and MPE obtained from 59 patients (Clinical details of the samples are provided in Table S1). The success rates were 44% (22/55) for LCOs and 80% (4/5) for normal lung tissues. The organoids retained the histological and genetic characteristics of original tumors (Fig. S1A-D). During biobanking, organoids derived from primary tumor tissues were capable of long-term culture under AO media conditions, whereas organoids from MPE and the SK-MES-1 cell line were observed to cease growth by the second passage (Fig. S1E). To address this issue, we introduced MPE supernatant into the AO culture system for MPE-derived organoids. With the support of MPE supernatant, these organoids could be successfully generated and maintained in a healthy state for at least three additional passages, demonstrating the facilitation of MPE on organoid formation. Moreover, the pathological features of organoids cultured with MPE remained consistent with the patient’s tumor tissue (Fig. S2).

We then investigated the impact of MPE on culture duration and drug sensitivity of LCOs. MPE was used to culture organoids generated from primary adenocarcinoma (L004T), metastatic adenocarcinoma (L004M) and squamous carcinoma (L016T). The results showed that MPE significantly promoted organoid proliferation, reducing the culture time by more than 50% (In AO media, diameter on the 9th day was 50–70 μm, whereas MPE group reached this size within only 2 to 4 days) (Fig. 1A-D). Our preliminary data also indicated that MPE supernatant consistently enhanced organoid growth, regardless of whether the MPE source was autologous or allogeneic, or whether the patients had undergone chemotherapy, with some cases showing more pronounced effects (Fig. 1E). We subsequently conducted drug sensitivity tests using Gemcitabine and Paclitaxel on four cases of LCOs cultured with MPE supernatant. MPE significantly increased LCO drug resistance to Gemcitabine in all cases and to PTX in one case (Fig. 1F).

**Fig. 1:**
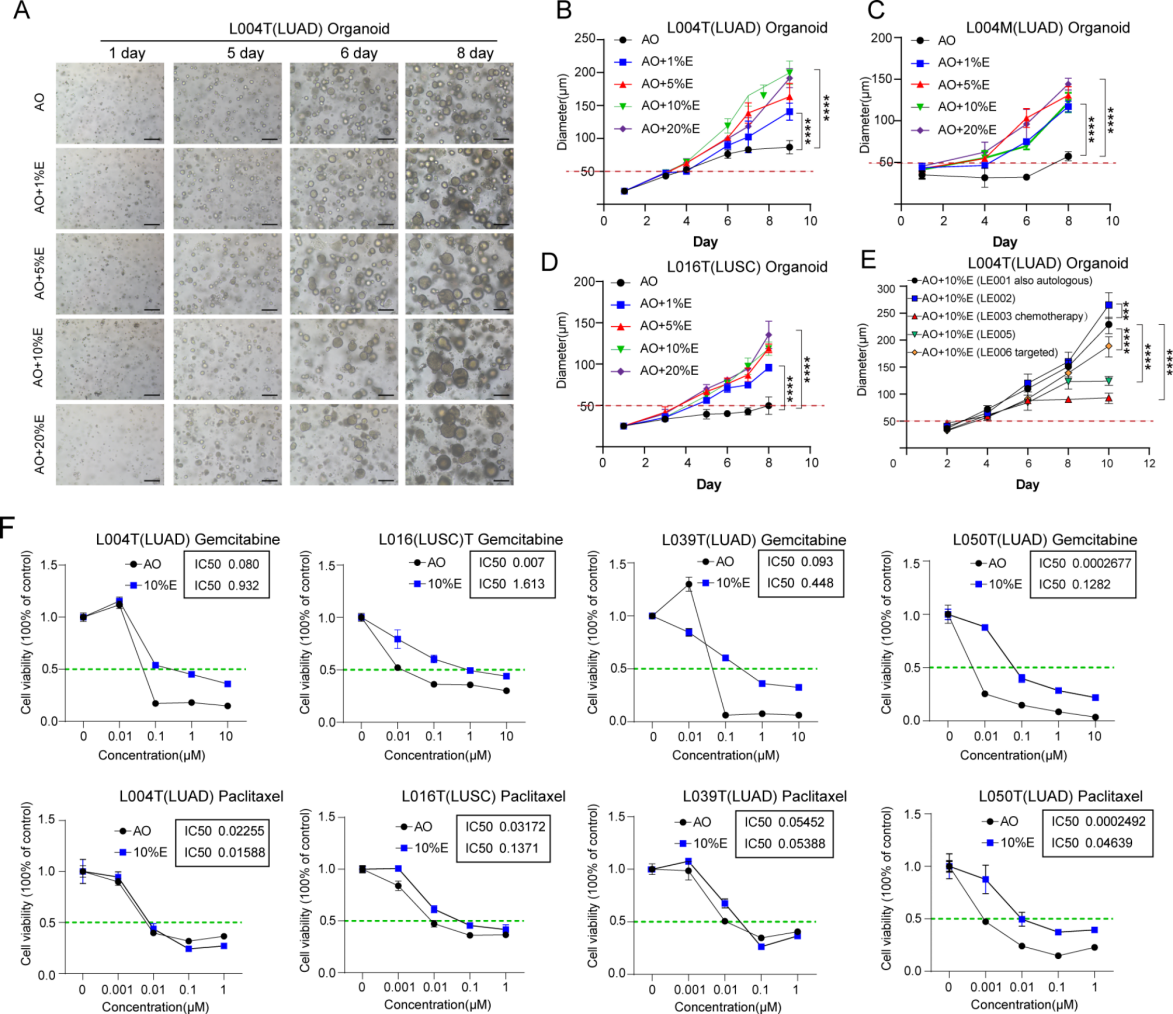
The effect of MPE on culture cycle and drug sensitivity of LCOs. (**A**) The bright-field images of L004T organoids cultured with or without MPE (LE001) supernatant. Black scale bars, 200 μm. (**B-D**) The statistics of L004T, L004M, L016T organoids’ diameters with different concentrations of MPE. (**E**) The proliferation effect of autologous and allogeneic MPE supernatant, without (LE002, LE005)/with (LE003, LE006) drug treatment on organoids. (**F**) The drug sensitivity of four cases tumor tissue-derived organoids with or without MPE supplement medium. The MPE supernatant was from L004 patients (LE001). *N* = 3, * *p* < 0.05, ** *p* < 0.01, *** *p* < 0.001, **** *p* < 0.0001

To elucidate the mechanism by which MPE influences organoids, particularly regarding drug resistance, we first explored changes in genetic characteristics and stem cells markers, and proliferation-related markers of LCOs with/without MPE support. The top 30 mutated genes in lung cancer were compared. All those mutations present in AO medium organoids were retained in MPE-treated organoids (Fig. 2A). The stem cell markers CD133 and SOX2 gradually concentrating towards the center and Ki67 was primarily expressed in the peripheral cells of organoids with MPE medium conditioning (Fig. 2B). These findings suggested that prolonged exposure to MPE does not alter the genetic makeup of the organoids but affects the distribution of stem cells and mature, differentiated cell subpopulations. We further examined the ultrastructure of organoids using transmission electron microscopy (TEM). After MPE treatment, the extracellular space was expanded (altering cell-cell adhesion). There was an increase in microvillus membranes and extracellular matrix, while the number of organelles such as mitochondria were less observed (Fig. 2C). Reduced cell-cell adhesion and extracellular matrix-mediated chemoresistance have been reported [9, 10]. Since these changes in cellular components and functions are closely linked to transcriptional regulation, RNA-seq was further used to investigate how MPE contributes to drug resistance in LCOs. Differentially expressed genes were analyzed using GO cellular component enrichment. The structural changes observed in the TEM analysis were reflected in the significantly enriched gene sets, including those related to extracellular space, extracellular matrix, and mitochondria (Fig. 2D). To further understand how MPE promotes organoid biobanking and influences tumor organoids, it is essential to identify the key components responsible for its effects. Our preliminary proteomic analysis reveals differences in proliferative effects among MPE samples, suggesting potential candidates for these active components (Fig. S3).

**Fig. 2:**
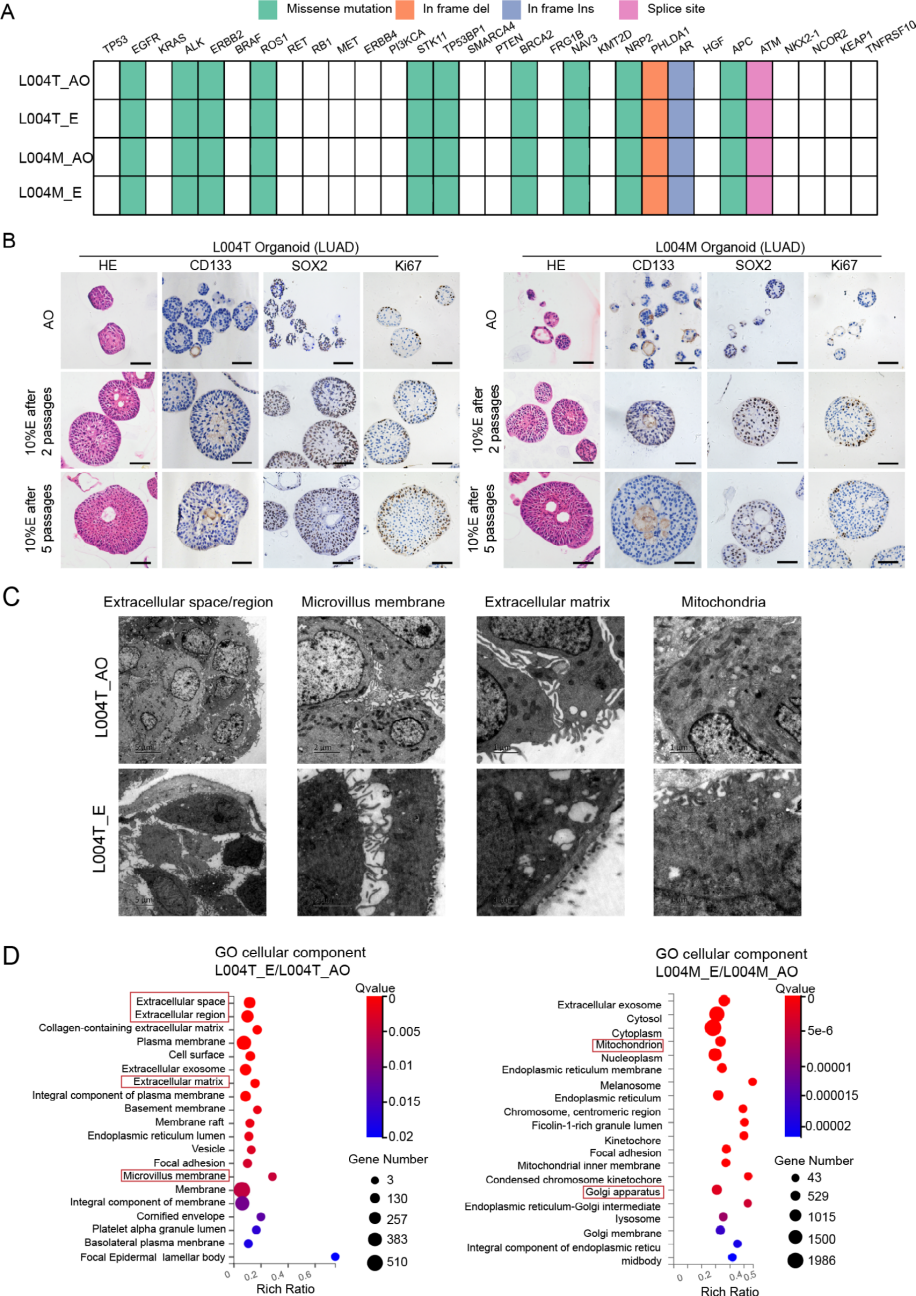
The mechanism of MPE biological effects on LCOs. (**A**) Heat-map analysis of the top 30 somatic mutations affecting cancer genes in L004T and L004M organoids cultured with/without MPE (LE001) supernatant. (**B**) IHC staining of proliferative and stemness related biomarkers. Black scale bar, 50 μm. (**C**) Representative microphotographs of transmission electron microscopy of L004T organoids with/without MPE culture, showing subcellular structure within organoids or surfaces. (**D**) The bubble chart depicts the top differentially expressed cell markers enriched in cellular component in organoids cultured with/without MPE. Colored bars represent q-value

Despite MPE improves maintenance of LCOs, it’s important to acknowledge potential limitations. MPE alters mRNA expression, raising concerns about representativeness of drug testing results. Further investigation is warranted to comprehensively assess the impact of MPE on drug response profiles. Our study has confirmed that five MPE samples promote organoid proliferation. However, the use of MPE samples from a single donor in majority of experiments may constrain the generalizability of our findings concerning the impact of MPE on organoid culture. Additionally, it is important to address the issue of normal airway basal cell contamination in LCO cultures under the current cultivation conditions, as highlighted by previous studies [11, 12]. In conclusion, incorporating MPE or its yet-to-be-identified active components into culture systems improves the efficiency of LCO establishment and may help to replicate aspects of the pleural tumor microenvironment, supporting the rapid generation of organoids for personalized drug testing.

## Electronic supplementary material

Below is the link to the electronic supplementary material.

Supplementary Material 1

Supplementary Material 2

Supplementary Material 3

## Data Availability

No datasets were generated or analysed during the current study.

## Abbreviations

LCOs: Lung cancer organoids
AO: Airway organoid
SCLC: Small cell lung cancer
NSCLC: Non-small cell lung cancer
MPE: Malignant pleural effusion
TEM: Transmission electron microscopy
CNAs: Copy number alterations
TTF-1: Thyroid transcription factor
CK7: Luminal epithelial marker cytokeratin 7
WES: Whole-Exome Sequencing
