# Supplementary material for: Malignant pleural effusion facilitates the establishment and maintenance of tumor organoid biobank with multiple patient-derived lung tumor cell sources
Source: Exp Hematol Oncol. 2024 Nov 15;13:115. doi: 10.1186/s40164-024-00581-9 (PMC11566167; doi:10.1186/s40164-024-00581-9)
Supplement: Supplementary file 3 — Supplementary Material 3 [file 40164_2024_581_MOESM3_ESM.docx]

**
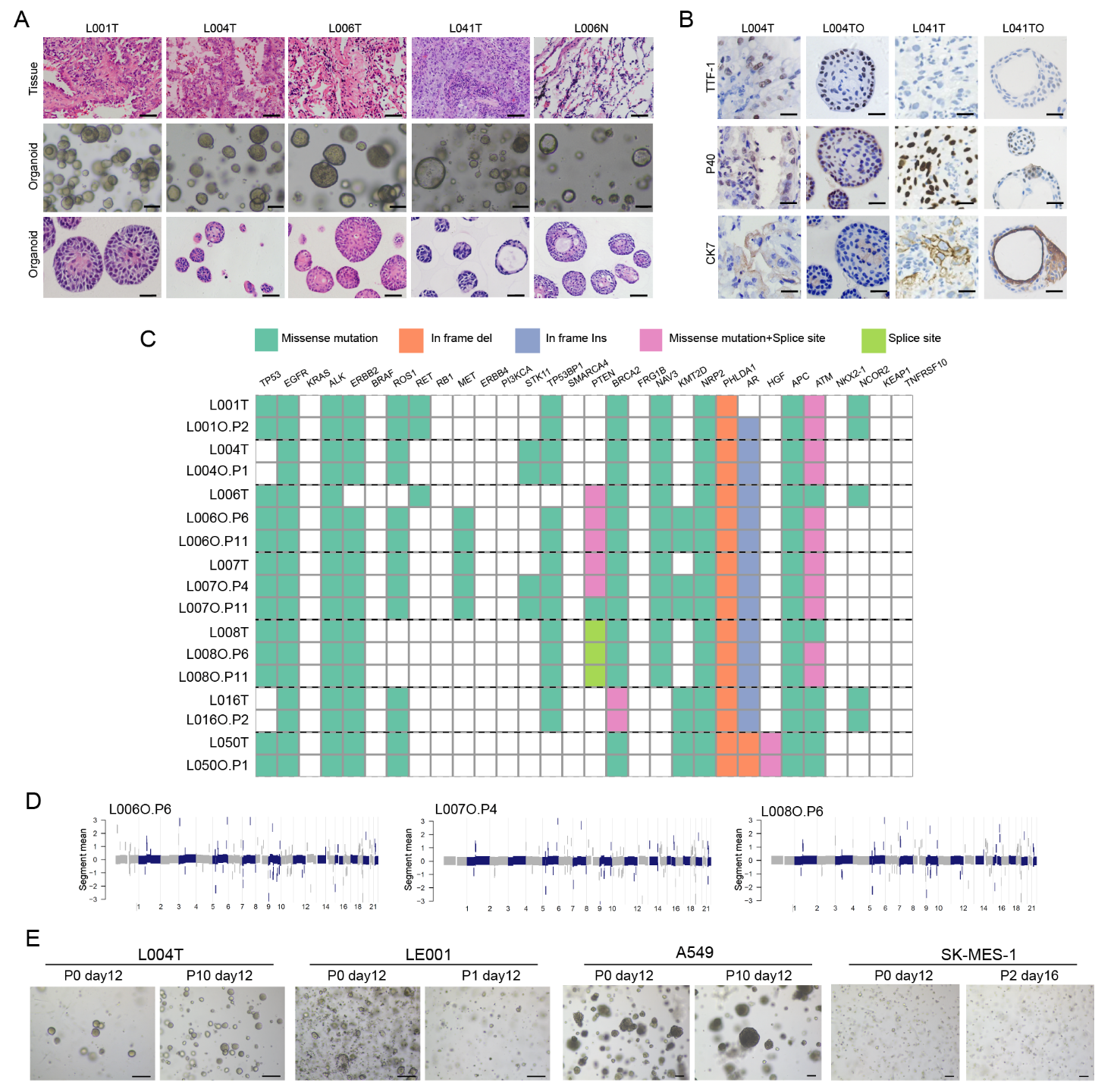
**

**Fig. S1** **Organoids recapitulate the histopathological and genetic signatures of their original tumors and some problematic samples failed within the second passages. A** Representative HE staining of tumor/normal lung tissues together with the bright-field images and corresponding HE staining of the organoids. Black scale bars of bright-field are 200 μm, HE and IHC are 50 μm. **B** Representative immunohistochemistry staining of TTF-1, P40, CK7 on lung tumors and corresponding organoids (T, primary tumors; O, organoids). Black scale bar, 25 μm. **C** Heat-map analysis of the top 30 somatic mutations affecting cancer genes in 7 LCO lines and their corresponding tissues. The letter O represents LCOs and T for patient tissue. **D** Examples of copy number profiles of organoids derived from tumor tissues show multiple gains and losses. **E** Most organoids can achieve long-term culture (L004T, A549). Organoids from MPE or some cell line cultured with AO media (LE001, SK-MES-1) were observed to drop out within the second passage.


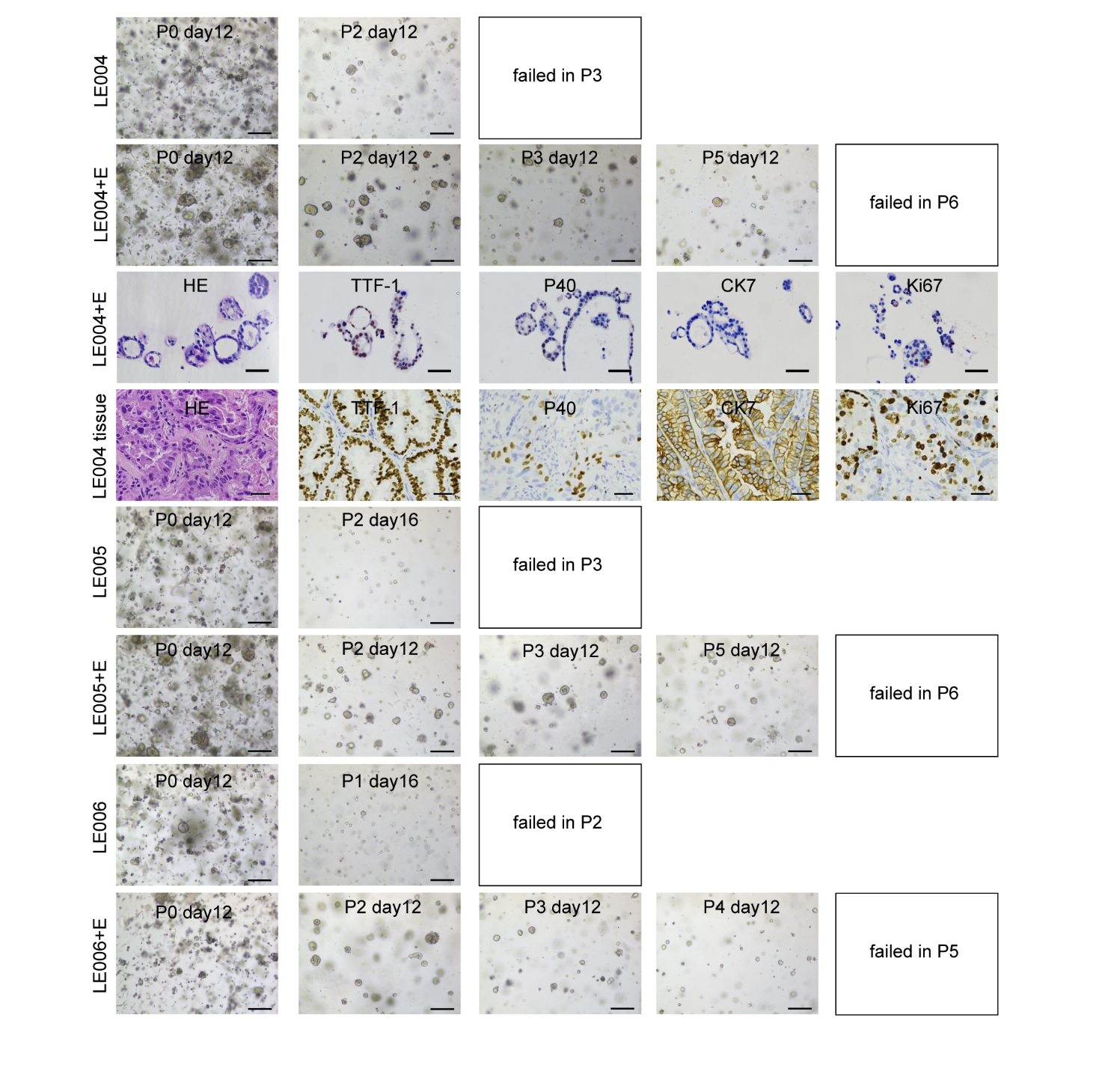


**Fig. S2** During the establishment and passage of organoids from three MPE samples (LE004, LE005, LE006), 10% MPE supernatant was added to AO medium. The organoids supported by MPE supernatant can extend in a good state at least 3 more passages. The pathological features of LE004+E organoids were consistent with the pathological diagnosis of surgical tissue. Black scale bar for bright field was 200 μm, and for pathological pictures 50 μm.


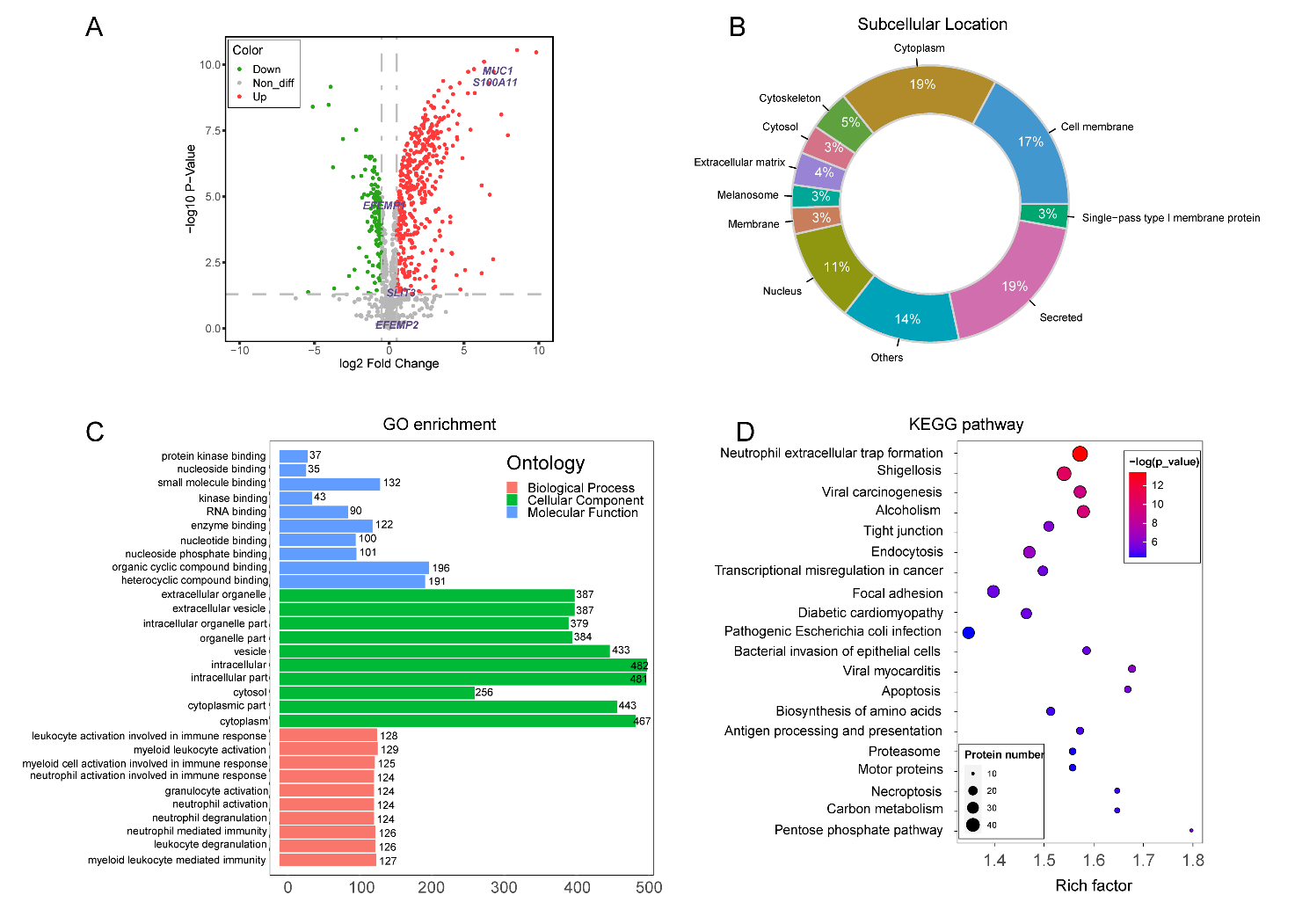


**Fig. S3 Identification and analysis of differentially expressed proteins (DEPs) in MPEs with different proliferative promoting effects. A** The volcanic map of DEPs level between LE003+LE005 MPE (weaker proliferative effects) and LE002+LE006 MPE (better proliferative effects). EGF related proteins SLIT3, EFEMP2 and EFEMP1 were indifferent expressed in both groups of MPE samples. EGF factors can promote rapid proliferation of organoids, which explains that all MPE samples can promote proliferation of organoids. MUC1 and S100A11 are proteins with higher differential expression. They are involved in tumor cell metabolism, proliferation, EMT and distant metastasis. This suggested the different levels of proteins that promote the malignant phenotype of tumors in different MPE samples lead to differences in their effectiveness in promoting organoids’ growth. **B** Subcellular localization of DEPs. 19% were secreted proteins, 19% located in the cytoplasm, 17% in the cell membrane. **C** Go enrichment analysis and functional annotation of DEPs between the two groups. **D** Pathway enrichment analysis of DEPs between the two groups.

**The supplementary Methods**

**Patient-derived lung tumor tissue and MPE collection**

Samples of lung cancer surgery (including paired normal tissues, if available), approximately 1~4 cm^3^ in volume, were obtained from the Affiliated Zhongshan Hospital of Dalian University. These samples were preserved in AdDF++++ (Advanced DMEM/F12 containing 1× Glutamax, 10 mM HEPES, 5 µM Y-27632, and Penicillin/Streptomycin) and transported directly to the laboratory following surgical removal. MPE samples were collected from patients either during surgery or who had been previously diagnosed with lung cancer. MPE samples were collected in 50 mL centrifuge tubes, typically totaling about 100~300 mL per sample. All participating patients provided informed consent, and the study was approved by the Ethics Committee of Affiliated Zhongshan Hospital of Dalian University (2022020–1). Clinical details of the samples are given in Table S1.

**Cell isolation and organoid culture**

*Cells from Tissues:*

Tumor or normal lung tissues were initially mechanically minced with scalpels until the volume was approximately 1 mm^3^. This was followed by digestion in AdDF++++ with 5 mg/mL collagenase II (Gibco, 17101015) and 100 µg/mL DNase I (sigma, DN25) solution for 12 h at 37 °C on a shaker at 140 rpm. After digestion, cell suspensions were filtered through a 70 µm cell strainer and then collected via centrifugation for 5 min at 1400 rpm. The cells were washed three times with D-PBS and then lysed in Red Blood Cell Lysis Buffer (Invitrogen, 2481406) for 5 min. Following this, the cells were resuspended in D-PBS, centrifuged, and counted. Subsequently, 5 × 10^4^ cells were embedded in 50 µL Matrigel (Corning, 356237) and plated in 50 µL droplets on a 24-well culture plate, which was inverted to solidify at 37 °C for 10 min following a 2-min incubation in a 37.5 °C water bath. After gelation, 500 µL of LCO medium (ingredients listed in Table S2) was added to the well. The media was refreshed every 2~3 days, and passaging occurred every two weeks.

*Cells from MPE:*

MPE samples in 50 mL centrifuge tubes were centrifuged at 1400 rpm to separate cells from fluid and plasma. The supernatant was filtered through a 0.22 µm filter (Millipore) and stored at –80 °C. Before addition, the MPE supernatant was centrifuged again for 5 min at 1400 rpm. The cell pellet was lysed with Red Blood Cell Lysis Buffer for 5 min. After removing the lysate, 1 × 10^5^ cells were resuspended in 50 µL Matrigel. Subsequent seeding and passaging procedures were consistent with tissue-derived organoid culture. Additional MPE culture involved mixing the previously filtered MPE supernatant with LCO medium according to a specified volume percentage for organoid culture.

*Lung cancer cell lines A549 and SK-MES-1:*

Cell lines in the exponential growth phase were digested with trypsin (Sigma, T4799), and 1.5 × 10^4^ cells were resuspended in 50 µL Matrigel. The subsequent culture method was consistent with the previously mentioned methods.

For the passaging process, tissue/MPE/cell lines-derived organoids were handled in the same manner. After removing the medium, 1.5 mL TrypLE (Gibco, 12604–021) was added to each well and incubated for 10 min at 37 °C for dissociation. Then, 1.5 mL AdDF++++ was added to halt digestion, followed by centrifugation at 1400 rpm for 5 min, and washing with AdDF++++. The cell pellets were resuspended in Matrigel, with an approximate splitting ratio of 1:2~1:4 every two weeks.

For cryopreservation, organoids were dissociated from the Matrigel with TrypLE into single cells with some aggregates. They were then frozen in Serum-Free Cell Freezing Medium (NCM Biotech, C40100) with Y-27632 (10 µM). The A549 and SK-MES-1 cell lines were generously provided by the Cell Bank/Stem Cell Bank, Chinese Academy of Sciences.

**Histology and Imaging**

Tissues were fixed with 4% paraformaldehyde overnight, followed by dehydration, paraffin embedding, and sectioning. Organoids embedded in Matrigel were washed with DPBS and fixed with 4% paraformaldehyde for 30 min. The organoids were then collected into a 1.5 mL EP tube, followed by centrifugation (5 min, 1400 rpm), DPBS washing, pre-embedding in agarose, fixed again with 4% paraformaldehyde overnight, dehydration, paraffin embedding, and sectioning.

For H&E staining, sections were stained with hematoxylin for 5 min, followed by a 10-min bluing step, and then stained with eosin for 5 min. For IHC staining, sections were placed in an antigen repair solution for 10 min in a microwave. Endogenous peroxidase was quenched using 3% hydrogen peroxide for 10 min at room temperature, followed by blocking with goat serum. Subsequently, sections were incubated with primary antibodies, including anti-napsin A (1:300; Santa Cruz, sc-517223), anti-TTF-1 (working fluid; MXB Biotechnologies, MAB-0599), anti-CK7 (1:500; Santa Cruz, sc-23876), anti-p40 (1:800; Santa Cruz, sc-5301), anti-CD133 (1:500; Cell Signaling Technology, 5860), and anti-Ki67 (1:500; CST, 9449). The sections were subsequently incubated with secondary antibodies (MXB Biotechnologies, KIT-9720) at room temperature and developed with a DAB chromogenic solution (ZSGB-BIO, ZLI-9018). The nuclei were counterstained with hematoxylin. Images were captured using an OLYMPUS BX53 microscope.

**Drug sensitivity test**

Organoids in the proliferating phase (about 14 days after passaging) were dissociated into single cells with some aggregates according to the passaging procedure described above. The individual cell pellets were collected and diluted to approximately 200 cells/µL with organoid medium containing 50% Matrigel (vol/vol). A 20 µL suspension was added to one well of a 96-well plate. After 3 days, concentration gradients of Gemcitabine and DMSO control were added. CellTiter-Glo3D reagent (Promega, G9681) was added after 6 days, with 5 min of shaking and a 25-min incubation at room temperature. Luminescence was measured using a SpectraMax microplate reader (Centro LB 963, Berthold). Data analysis was performed using GraphPad Prism 8, and IC50 values were determined manually. Cell viability of drug treated group was normalized using the drug untreated organoid with the same medium (AO or 10% MPE) as control.

**Transmission electron microscopy**

PDOs cultured for two passages, with diameters ranging from 70 to 150 µm, were fixed for 24 h at 4 °C in cacodylate-buffered 4% glutaraldehyde, post-fixed in 1.5% osmium tetroxide dissolved in cacodylate buffer. The samples were then dehydrated in an ascending scale of ethyl alcohol and embedded in Epon. Ultra-thin slices were stained with uranyl acetate and lead citrate. Prior to ultrastructural characterization of LCOs, all samples were initially observed to identify morphological features useful for analysis. Subsequently, samples were analyzed using a Tecnai Spirit Biotween 120KV EP5018/40 electron microscope. Intracellular lumens, extracellular space, nuclei were studied at 2000~2500 magnification. Mitochondria, lysosomes, secretion granules, and other organelles were observed at 6000 magnifications.

**DNA extraction and whole-exome sequencing analysis**

Genomic DNA was extracted from both organoids and matched formalin-fixed & paraffin-embedded (FFPE) primary tumor tissues using the MagPure FFPE DNA/RNA Kit (#IVD3026, Angen Biotech, China). WES libraries were sequenced using paired-end (2 × 100 bp) runs on the BGISEQ-500 platform, achieving depths of ~250× (~33 Gb per sample). The WES data was aligned to the human reference genome GRCh37 with the Burrows–Wheeler Aligner v0.7.17, using the default options and further processed by the Genome Analysis Toolkit (GATK). This includes marking duplicate reads and performing base quality recalibration, as well as filtering the original variant calling results to select mutations with variant allele frequency (VAF) greater than 5% and total reads in the sample greater than 20. After obtaining a highly reliable and high-quality mutation set, Annodb was used to annotate the mutation results. Copy number variations (CNV) detection and visualization were performed using CNVkit. Mutations were then compared with known driver genes from databases and literatures to identify known driver genes. The proportions of SNPs and types of base substitutions were counted, and variations in tumor tissues and matched organoids were visualized using Venn plots.

**RNA sequencing and analysis**

RNA was isolated from organoids with/without MPE cultured using the RNeasy Plus Mini Kit (QIAGEN) according to the manufacturer’s instructions. The RNA libraries were sequenced with paired-end 150 bp reads on the BGISEQ platform. Raw reads obtained from sequencing were filtered using SOAPnuke. The clean reads were then aligned to the reference sequence GRCh37 through HISAT. Subsequently, gene quantitative analysis and various analyses based on gene expression levels (principal component analysis, correlation analysis, differential gene screening, etc.) were conducted. Differentially expressed genes among the selected samples were explored and analyzed through GO functional significance enrichment analysis and pathway significance enrichment analysis.

**Proteomic analysis**

*Protein extraction and digestion for LC-MS/MS*

LE002 and LE006, LE003 and LE005 were 1:1 (v/v) mixed respectively, three parallel tests were performed. 800 μL TCA-acetone was added to 200 μL sample for deproteinization. 8M Carbamide, 30mM HEPES, 1m PMSF, 2mM EDTA, 10 mM DTT buffer was used for sample lysis and protein extraction. The amount of protein was quantified using the BCA Protein Assay Kit (Bio-Rad, Hercules, CA, USA). Protein digestion by trypsin was performed using the filter-aided sample preparation (FASP) procedure.

*LC-MS/MS analysis*

LC-MS/MS analysis was performed using the Vanquish™ Neo UHPLC (Thermo, USA) coupled to OrbitrapExploris 480 mass spectrometer (Thermo Scientific) for 80 min. The peptides were loaded onto a C18 analytical column (20 cm long, 75 mm inner diameter) in buffer A (0.1% formic acid) and separated at a linear gradient of buffer B (80% acetonitrile and 0.1% formic acid) at a flow rate of 300 nL/min. The mass spectrometer was operated in DIA mode. The mass spectrometer recorded ion mobility MS spectra over a mass range of m/z 398–1002 and scanning resolution 120000.

*Bioinformatic analysis of proteomics data*

The raw DIA data for each sample were combined and searched using DIANN software for identification and quantitation analysis. Based on quantitative results, t-tests were performed on all groups to differentially expressed proteins (DEPs). The protein sequences of the DEPs were locally searched using NCBI BLAST, client software (ncbi-blast-2.2.28+-win32.exe) and InterProScan to find homologous sequences, then GO terms were mapped. For KEGG analysis, the DEPs were BLASTed against the online KEGG database (http://geneontology.org/) to retrieve their KEGG orthology identifications and were mapped pathways.

**Statistical analysis**

Experiments in this study were based on the analysis of at least three replicates. Statistical analyses were performed using Excel 2019 and GraphPad Prism 8 (Version X). P values were calculated by Student’s t-test, and error bars represent ±SD. A *p*-value < 0.05 was considered statistically significant (* *p* < 0.05, ** *p* < 0.01, *** *p* < 0.001, **** *p* < 0.0001).

| **Table S1.** List of lung samples and matched organoids in this study. | | | | | | | | | | | |
| --- | --- | --- | --- | --- | --- | --- | --- | --- | --- | --- | --- |
| **Case** | **Sample** | **Sex** | **Age** | **Lung cancer type** | **subtype** | **stage** | **Sample type** | **Organoid formation** | **Thawing test** | **Medication**  **or not** | **Passages** |
| 1 | L001T | F | 70 | Adenocarcinoma | Acinar | pT1bN0M0/IA2 | Surgical specimen | ○ | ○ | × | 20 |
| 2 | L001N | F | 70 | - | - | - | Surgical specimen | ○ | × | × | 7 |
| 3 | L002T | F | 68 | Adenocarcinoma | Solid 60%；  Acinar 40% | sT2aN0M0/IB | Surgical specimen | × | × | × | 2 |
| 4 | L003T | F | 58 | Adenocarcinoma | Solid | sT2aN0M0/IB | Surgical specimen | ○ | ○ | × | 11 |
| 5 | L004T | F | 78 | Adenocarcinoma | Acinar 80%；  Papillary 20% | sT2aNxM1c/IVB | Surgical specimen | ○ | ○ | × | 14 |
| 6 | L004M | F | 78 | Adenocarcinoma | - | - | Surgical specimen | ○ | ○ | × | 12 |
| 7 | L004N | F | 78 | - | - | - | Surgical specimen | ○ | ○ | × | 6 |
| 8 | LE001 | F | 78 | Adenocarcinoma | - | sT2aNxM1c/IVB | Surgical specimen | ○ | × | × | 4 |
| 9 | L005T | F | 50 | Metastatic renal clear cell carcinoma | - | - | Surgical specimen | × | × | × | 3 |
| 10 | L006T | F | 50 | Infiltrating adenocarcinoma | Acinar 90%；Adherent 10% | pT1bN0M0/IA2 | Surgical specimen | ○ | ○ | × | 13 |
| 11 | L006N | F | 50 | - | - | - | Surgical specimen | ○ | ○ | × | 13 |
| 12 | L007T | M | 65 | Adenocarcinoma | Acinar 90%；Adherent 10% | pT1bN0M0/IA2 | Surgical specimen | ○ | ○ | × | 12 |
| 13 | L007N | M | 65 | - | - | - | Surgical specimen | ○ | ○ | × | 12 |
| 14 | L008T | M | 65 | Adenocarcinoma | Solid | sT1bN0M0/IA2 | Surgical specimen | ○ | ○ | × | 13 |
| 15 | L008N | M | 65 | - | - | - | Surgical specimen | ○ | ○ | × | 13 |
| 16 | L009T | F | 43 | Adenocarcinoma | Acinar | pT1aN0M0/IA1 | Surgical specimen | × | × | × | 2 |
| 17 | L010T | M | 64 | Adenocarcinoma | Adherent | sT1aN0M0/IA1 | Surgical specimen | × | × | × | 2 |
| 18 | L011T | F | 68 | Adenocarcinoma | Papillary | sT1bN0M0/IA2 | Surgical specimen | ○ | × | × | 6 |
| 19 | L012T | F | 48 | Adenocarcinoma | Acinar | pT1bN0M0/IA2 | Surgical specimen | × | × | × | 2 |
| 20 | L013T | F | 67 | Adenocarcinoma | Adherent | pT1bN0M0/IB | Surgical specimen | × | × | × | 2 |
| 21 | L014T | M | 66 | Adenocarcinoma | Acinar 50%；  Solid 50% | pT1cN0M0/IA3 | Surgical specimen | × | × | × | 2 |
| 22 | L015T | M | 65 | Adenocarcinoma | Acinar 90%；Adherent 10% | sT1aN0M0/IA1 | Surgical specimen | × | × | × | 2 |
| 23 | LE002 | M | 79 | Adenocarcinoma | Acinar | cT4NxM1/IV | Pleural effusion | ○ | × | × | 4 |
| 24 | L016T | F | 73 | Squamous | - | pT1cN1M0/IIB | Surgical specimen | ○ | ○ | × | 13 |
| 25 | L017T | M | 71 | Adenocarcinoma | - | pT1bN0M0/IA2 | Surgical specimen | × | × | × | 2 |
| 26 | L018T | F | 62 | Adenocarcinoma | - | sT1aN0M0/IA1 | Surgical specimen | × | × | × | 2 |
| 27 | L019T | M | 62 | Adenocarcinoma | - | sT1bN0M0/IA | Surgical specimen | × | × | × | 2 |
| 28 | L020T | F | 61 | LCNEC | - | sT3N0M0/IIB | Surgical specimen | × | × | × | 2 |
| 29 | L021T | F | 64 | Adenocarcinoma | Acinar | sT1bN0M0/IA2 | Surgical specimen | × | × | × | 2 |
| 30 | L022T | F | 71 | Adenocarcinoma | Acinar | sT2aN0M0/IB | Surgical specimen | × | × | × | 2 |
| 31 | L023T | M | 29 | Adenocarcinoma | - | sT1aN0M0/IA | Surgical specimen | × | × | × | 2 |
| 32 | L024T | F | 74 | Adenocarcinoma | Adherent | cT1bN0M0/IA2 | Surgical specimen | × | × | × | 2 |
| 33 | LE003 | M | 40 | Adenocarcinoma | - | cTxN2M1 | Pleural effusion | ○ | × | ○ | 3 |
| 34 | L025T | M | 65 | Adenocarcinoma | Acinar 65%；Solid 30%；Adherent 5% | sT2aNOMO/Ib | Surgical specimen | ○ | ○ | × | 13 |
| 35 | L026T | F | 68 | Adenocarcinoma | Acinar | sT1aN0M0/IA | Surgical specimen | ○ | × | × | 6 |
| 36 | L027T | M | 68 | Adenocarcinoma | Acinar | sT1bN0M0/IA2 | Surgical specimen | ○ | × | × | 4 |
| 37 | LE004+E | M | 82 | Adenocarcinoma | - | sT2aN0M1a/IV | Pleural effusion | ○ | × | ○ | 6 |
| 38 | L028T | F | 49 | Adenocarcinoma | Acinar | sT1bN0M0/IA2 | Surgical specimen | ○ | × | × | 6 |
| 39 | L029T | F | 75 | Adenocarcinoma | Acinar 80%；Solid 15%；Adherent 5% | sT1bN0M0/IA2 | Surgical specimen | ○ | × | × | 4 |
| 40 | L030T | M | 76 | AAH | - | - | Surgical specimen | ○ | × | × | 8 |
| 41 | L031T | F | 60 | Adenocarcinoma | Acinar+Papillary | sT1cN0M0/IA3 | Surgical specimen | ○ | × | × | 4 |
| 42 | LE005+E | F | 81 | Adenocarcinoma | - | - | Pleural effusion | ○ | × | ○ | 6 |
| 43 | L032T | F | 56 | Adenocarcinoma | Solid | sT1bN0M0/IA | Surgical specimen | ○ | × | × | 7 |
| 44 | L033T | M | 55 | Adenocarcinoma | Acinar | sT2aN0M0/IIIA | Surgical specimen | ○ | × | × | 7 |
| 45 | L034T | M | 69 | Adenocarcinoma | Acinar | sT1bN0M0/IA | Surgical specimen | ○ | × | × | 9 |
| 46 | L035T | F | 59 | Adenocarcinoma | Acinar | sT1bN0M0/IA2 | Surgical specimen | ○ | × | × | 4 |
| 47 | L036T | M | 78 | Adenocarcinoma | Acinar | sT2aN0M0/IB | Surgical specimen | × | × | × | 2 |
| 48 | L037T | F | 59 | Adenocarcinoma | Adherent | sT1aN0M0/IA2 | Surgical specimen | × | × | × | 2 |
| 49 | LE006+E | F | 64 | Adenocarcinoma | - | cT4N3M1 | Pleural effusion | ○ | × | ○ | 5 |
| 50 | L038T | M | 76 | Squamous | - | - | Surgical specimen | × | × | × | 2 |
| 51 | L039T | F | 46 | Adenocarcinoma | Mucinous | sT1bN0M0/IA | Surgical specimen | ○ | ○ | × | 12 |
| 52 | L040T | F | 71 | Adenocarcinoma | - | sT2aN0M0/IB | Surgical specimen | × | × | × | 2 |
| 53 | L041T | M | 58 | Squamous | - | - | Surgical specimen | ○ | ○ | × | 8 |
| 54 | L042T | F | 77 | Adenocarcinoma | - | sT2aN0M0/IB | Surgical specimen | ○ | ○ | × | 6 |
| 56 | L043T1 | F | 64 | Adenocarcinoma | - | pT1bN0M0/IA2 | Surgical specimen | ○ | ○ | × | 6 |
| 57 | L043T2 | F | 64 | Adenocarcinoma | - | pT1bN0M0/IA2 | Surgical specimen | ○ | ○ | × | 6 |
| 58 | L044T | F | 67 | Adenocarcinoma | Acinar | sT1bN0M0/IA2 | Surgical specimen | ○ | ○ | × | 6 |
| 59 | L045T | F | 53 | Adenocarcinoma | - | - | Surgical specimen | × | × | × | 2 |
| 60 | L046T | F | 50 | Adenocarcinoma | Adherent | sT1bN0M0/IA2 | Surgical specimen | ○ | ○ | × | 5 |
| 61 | L047T | M | 62 | Adenocarcinoma | - | sT1bN0M0/IA2 | Surgical specimen | × | × | × | 2 |
| 62 | L048T | F | 64 | Adenocarcinoma | - | sT1bN0M0/IA2 | Surgical specimen | ○ | ○ | × | 6 |
| 63 | L049T | F | 30 | Adenocarcinoma | Solid 70%；Acinar 30%； | sT2aN0M0/IB | Surgical specimen | ○ | ○ | × | 8 |
| 64 | L050T | M | 82 | Adenocarcinoma | Acinar 60%；Complex glandular structure 20%；Solid 15%；Micropapillary 5% | pT2aN2M0/IIIA | Surgical specimen | ○ | ○ | × | 5 |
| 65 | L051T | M | 68 | Adenocarcinoma | Acinar | pT1cN0M0/IA3 | Surgical specimen | ○ | ○ | × | 8 |
| 66 | L052T | F | 66 | Adenocarcinoma | Acinar 90%；Adherent10% | pT1cN0M0/IA3 | Surgical specimen | ○ | ○ | × | 5 |
| 67 | L053T | M | 66 | Adenocarcinoma | - | sT1cN0M0/IA | Surgical specimen | ○ | ○ | × | 6 |
| T tumor tissue, N normal lung tissue, M metastatic lung cancer, E effusion，○ succeed, × fail | | | | | | | | | | | |

| **Table S2. Lung cancer organoid medium recipe** | | | |  |  |
| --- | --- | --- | --- | --- | --- |
|  |  |  |  |  |  |
| Medium component | Supplier | Catalogue number | stock concentration | Final concentration | 50ml media |
| R-Spondin 1 | Peprotech | 120-38 | 50ug/ml,200x | 250ng/ml | 250ul |
| FGF 7 | Peprotech | 100-19 | 25ug/ml,1000x | 25 ng/ml | 50ul |
| FGF 10 | Peprotech | 100-26 | 20ug/ml,1000x | 20 ng/ml | 50ul |
| Noggin | Peprotech | 120-10C | 100ug/ml,1000x | 100 ng/ml | 50ul |
| A83-01 | Tocris | 2939 | 25mM,50000x | 500 nM | 1ul |
| Y-27632 | Selleck | S1049 | 5mM,1000x | 5 mM | 50ul |
| SB202190 | Selleck | S1077 | 10mM,20000x | 500 nM | 2.5ul |
| B27 supplement | Gibco | 17504-44 | 50x | 1x | 1ml |
| N-Acetylcysteine | Sigma | A9165 | 500mM,400x | 1.25 mM | 125ul |
| Nicotinamide | Sigma | N0636 | 2M,200x | 5 mM | 250ul |
| GlutaMax 100x | Gibco | 35050-061 | 100x | 1x | 500ul |
| Hepes | Sigma | H4034 | 1M,100x | 10 mM | 500ul |
| Primocin | Invivogen | Ant-pm-1 | 50mg/ml,1000x | 50 mg/ml | 50ul |
| Penicillin/Streptomycin | Sigma | V900929 |  | 100 U·ml^-1^ / 100 mg·ml^-1^ | 500ul |
| Advanced DMEM/F12 | Gibco | 12634010 |  | 1x |  |
